# White Matter Plasticity in Anxiety: Disruption of Neural Network Synchronization During Threat-Safety Discrimination

**DOI:** 10.3389/fncel.2020.587053

**Published:** 2020-11-05

**Authors:** Jia Liu, Ekaterina Likhtik, A. Duke Shereen, Tracy A. Dennis-Tiwary, Patrizia Casaccia

**Affiliations:** ^1^Advanced Science Research Center at the Graduate Center, Neuroscience Initiative, City University of New York, New York, NY, United States; ^2^Department of Biology, Hunter College, City University of New York, New York, NY, United States; ^3^Graduate Program in Biology at the Graduate Center, City University of New York, New York, NY, United States; ^4^Department of Psychology, Hunter College, City University of New York, New York, NY, United States; ^5^Graduate Program in Psychology and Behavioral and Cognitive Neuroscience at the Graduate Center, City University of New York, New York, NY, United States; ^6^Graduate Program in Biochemistry at the Graduate Center, City University of New York, New York, NY, United States; ^7^Department of Neuroscience, Icahn School of Medicine at Mount Sinai, New York, NY, United States

**Keywords:** myelin, synchrony, oligodendroccyte, connectivity, interneuron

## Abstract

Recent evidence highlighted the importance of white matter tracts in typical and atypical behaviors. White matter dynamically changes in response to learning, stress, and social experiences. Several lines of evidence have reported white matter dysfunction in psychiatric conditions, including depression, stress- and anxiety-related disorders. The mechanistic underpinnings of these associations, however, remain poorly understood. Here, we outline an integrative perspective positing a link between aberrant myelin plasticity and anxiety. Drawing on extant literature and emerging new findings, we suggest that in anxiety, unique changes may occur in response to threat and to safety learning and the ability to discriminate between both types of stimuli. We propose that altered myelin plasticity in the neural circuits underlying these two forms of learning relates to the emergence of anxiety-related disorders, by compromising mechanisms of neural network synchronization. The clinical and translational implications of this model for anxiety-related disorders are discussed.

## Introduction

Myelination in vertebrates, represents a successful mechanism of adaptation to the development of complex behaviors, requiring increased speed of axonal conduction. While in some invertebrates fast transmission is achieved by decreasing resistance due to axonal expansion, in craniates and jawed fish the insulation provided by myelin allows axons of similar caliber to increase their speed of communication by several hundred folds (Tomassy et al., 2016). Oligodendrocytes (OLs) are the myelin-forming cells of the central nervous system (CNS). They derive from oligodendrocyte progenitor cells (OPCs), which continue to proliferate and differentiate into new OLs throughout life (Dimou et al., 2008; Zhu et al., 2011; Young et al., 2013; Hill et al., 2018). The insulating properties of myelin are essential for saltatory axonal conduction. However, in higher-order organisms, the myelin sheath is not a static cellular compartment but is rather a dynamic membrane, capable of providing metabolic supports to axons in conditions of elevated energetic demands (Nave, 2010; Saab and Nave, 2017). Finally, recent findings have defined the formation and remodeling of new myelin in response to experience and learning as key contributors to physiological brain function and behavior (Liu et al., 2012; Makinodan et al., 2012; Gibson et al., 2014; Mckenzie et al., 2014; Hughes et al., 2018; Mitew et al., 2018; Bonnefil et al., 2019; Geraghty et al., 2019; Swire et al., 2019; Pan et al., 2020; Steadman et al., 2020). Yet, the functional significance of myelinating OLs concerning psychological adaptation remains poorly understood. Here, we first discuss the concept of myelin plasticity and review evidence that it occurs in response to social experiences and in the context of learning of motor and non-motor skills, including learning about threat. We then review literature related to white matter alterations in psychiatric disorders, with a focus on stress- and anxiety-related disorders. Finally, we discuss emerging evidence supporting associations between altered myelin plasticity in circuits regulating threat and safety learning and the ability to discriminate between threat and safety stimuli as purported anxiogenic mechanisms and outline key clinical and translational implications.

## White Matter and Myelin Plasticity in Response to Social Experience Or Learning

The concept of myelin plasticity includes diverse types of cellular processes such as *de novo* myelin formation and remodeling of pre-existing myelin. *De novo* myelination refers to the differentiation of local resident OPCs into myelin-forming OLs and/or wrapping of previously unmyelinated axons or axonal segments (Tomassy et al., 2014; Hill et al., 2018). Myelin remodeling refers to changes in the number of wraps around myelinated axons or in the length of myelinated segments between two nodes of Ranvier (i.e., internodal length).

Myelin plasticity was initially reported in studies addressing exposure to social stressors both in humans and in animal models. One of the initial studies conducted in human children exposed to severe childhood neglect identified reduced thickness of the corpus callosum area in these individuals compared to controls (Teicher et al., 2004; Mehta et al., 2009). Maternal deprivation in rodents, early weaning, and social deprivation during the critical period of adolescence also resulted in defective myelination detected in juvenile mice (Kodama et al., 2008; Makinodan et al., 2012; Yang et al., 2017). The effect of social stress was not limited to a critical developmental period, as adult mice exposed to chronic variable stress, social isolation or social defeat, also altered the OL transcriptome, and decreased myelin thickness in the medial prefrontal cortex (mPFC; Liu et al., 2012, 2018; Bonnefil et al., 2019). Together, these studies provide clear evidence of a link between myelination of specific brain regions and social experience.

White matter plasticity was reported to be modulated also in response to motor and non-motor learning. Myelination of distinct neural pathways in children, for instance, follows a stereotyped sequence that coincides with the development of important motor milestones, such as sitting, crawling, and then walking (Aubert-Broche et al., 2008; Tomassy et al., 2016). Learning how to juggle in adulthood increased myelination of subcortical white matter at the right posterior intraparietal sulcus, which was detected as increased fractional anisotropy (FA) in MRI (Scholz et al., 2009). The inverse relationship between the extent of white matter changes and age of training was assessed by the analysis of FA at the posterior midbody/isthmus of the corpus callosum in piano players, which revealed greater connectivity and sensorimotor synchronization performance in those who learned earlier rather than later in life (Steele et al., 2013). Non-motor learning was similarly associated with changes in white matter. For example, in children aged 3 months to 4 years, the myelin volume fraction in the frontal and temporal cortices showed a positive correlation with predicted language abilities, which strengthened with age (O’Muircheartaigh et al., 2014). In subjects learning a second language as adults, systematic, learning-dependent changes were also observed in the white matter tracts associated with traditional left hemisphere language areas and their right hemisphere analogs (Schlegel et al., 2012).

Animal studies repeatedly demonstrate life-long myelin plasticity in response to motor and non-motor learning. For instance, learning a novel motor skill in rats resulted in higher FA in the subcortical white matter of the sensorimotor cortex and increased myelin protein expression after training (Sampaio-Baptista et al., 2013). The necessity of myelin plasticity for skill acquisition and memory consolidation was further demonstrated using transgenic mice. Impairing new myelin synthesis by conditional ablation of the lineage-specific transcription factor *Myrf*, prevented *de novo* myelination during training and impaired new motor skill acquisition while retaining intact general motor function (Mckenzie et al., 2014). Similarly, preventing the formation of new OLs and myelin impaired spatial memory formation and water maze performance (Pan et al., 2020; Steadman et al., 2020).

Additional studies support a role for OL lineage cells in memory consolidation. Impaired formation of new myelin *via* lineage-specific ablation of the transcription factor *Myrf* did not affect contextual freezing immediately after learning, but rather impaired memory retrieval (Pan et al., 2020; Steadman et al., 2020), thereby suggesting that generation of new OLs was required for fear memory consolidation. It was also reported that memory consolidation required the occurrence of rhythmic oscillatory communication to synchronize activity across brain regions (Pajevic et al., 2014), which was impaired in the *Myrf* conditional knockout mice (Steadman et al., 2020), thereby highlighting the functional relevance of myelination for learning-induced synchronized activity.

## Myelin and White Matter Alterations in Stress- and Anxiety-Related Disorders

**Figure 1:**
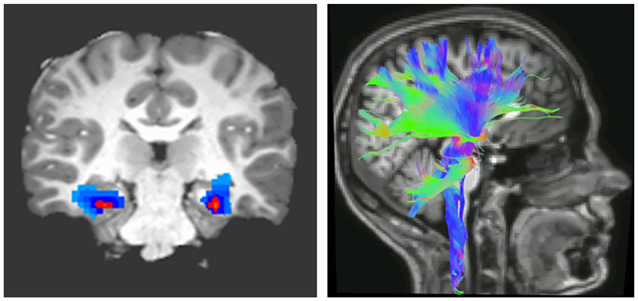
Imaging brain connectivity. Two widely used imaging techniques to assess connectivity are functional magnetic resonance (fMRI; left) and diffusion tensor imaging (DTI; right). fMRI indirectly measures neural activity between two or more regions through statistical analysis of correlated changes in the blood oxygen level-dependent MRI signal (Buxton, 2009). In contrast, DTI measures structural connectivity or the organization of white matter tracts running between neural regions. Fractional anisotropy (FA), derived from DTI, indicates the degree to which water molecules preferentially diffuse along one direction. Because myelin restricts water molecules to diffuse mainly along the direction of axonal bundles, higher FA values are often interpreted as indicating greater myelination or organization of white matter tracts (Thomason and Thompson, 2011).

The importance of myelin plasticity in response to external conditions and shaping behavioral consequences led to the concept that myelination of white matter tracts regulating learning about threat may be altered in pathologies characterized by behavioral maladaptation and are associated with changes in brain function. Magnetic resonance imaging (MRI) allows for the indirect measurement of brain connectivity, both functionally (fMRI) and structurally (diffusion MRI; Figure 1). Parameters that are characteristically measured are FA, which indicates the degree to which water molecules preferentially diffuse along one direction. Because myelin restricts water molecules to diffuse mainly along the direction of axonal bundles, higher FA values are often interpreted as indicating greater myelination or organization of white matter tracts (Thomason and Thompson, 2011). Complementary diffusion values, such as radial, axial, and mean diffusivities, provide additional information related to the integrity, caliber, and myelination of white matter (Song et al., 2003).

**Figure 2:**
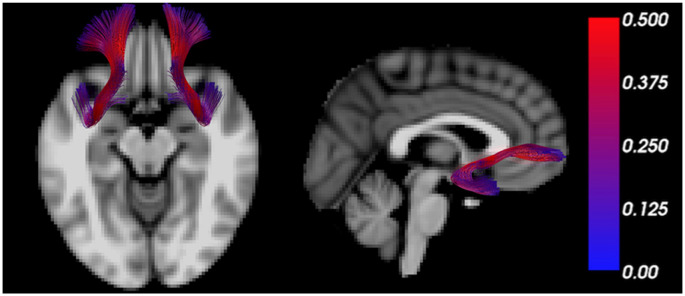
The uncinate fasciculus is a characteristic trajectory connecting the prefrontal cortex (PFC) and amygdala. MRI tractography depicts the uncinate fasciculus overlain on transverse (left) and sagittal (right) T1 weighted sections. The color bar scales the tract by fractional anisotropy (FA) values.

The uncinate fasciculus is an important white matter tract connecting brain regions regulating the threat response (e.g., amygdala) with those regulating behavior (e.g., PFC; Figure 2), and its myelination follows a characteristic developmental trajectory during adolescence, reaching stability in young adulthood (Lebel and Beaulieu, 2011; Thomason and Thompson, 2011). Early in life, excitatory signals have been shown to emerge from the amygdala directed to the PFC, while later in life, inhibitory signaling from the PFC to the amygdala favors emotional regulation (Ghashghaei et al., 2007; Cressman et al., 2010). While functional connectivity studies do not indicate the direction of influence, a valence switch from positive to negative PFC-amygdala fMRI correlations during normal development supports the existence of this developmental pattern (Gee et al., 2013).

The progressive increase of FA in the uncinate fasciculus, in young healthy subjects, reflects effective myelination of this tract and results in facilitated communication between PFC and amygdala (Kim and Whalen, 2009; Tromp et al., 2012). In contrast, decreased FA in the uncinate fasciculus was reported in subjects with anxiety-related traits and was suggestive of disrupted or inefficient myelination (Westlye et al., 2011). Importantly, a reduction in structural connectivity, as indicated by reduced bilateral FA in both uncinate fasciculi was consistently detected among individuals with a generalized anxiety disorder (Tromp et al., 2012), and also in children exposed to socioemotional deprivation (Eluvathingal et al., 2006). Decreased FA and radial diffusivity were also detected in the left uncinate fasciculus of affected monozygotic adolescent twins with anxiety disorders compared to unaffected siblings (Adluru et al., 2017), collectively suggesting compromised communication between PFC and amygdala in such individuals (Yavas et al., 2019).

While altered connectivity between the amygdala and PFC has been well documented in several stress and anxiety-related disorders (Tromp et al., 2012), pathological inflammatory demyelination of the septo-fornical area, has also been reported in multiple sclerosis patients with high anxiety, and suggested to contribute to its pathogenesis (Palotai et al., 2018).

However, the alteration of white matter microstructure involved in anxiety-related personality traits is not restricted to corticolimbic pathways. For example, harm avoidance in adult subjects was positively associated with radial and mean diffusivity, not only in the uncinate fasciculus but also in the anterior thalamic radiation, corpus callosum, parahippocampal cingulum, corticospinal tract, and inferior and superior longitudinal fasciculi (Westlye et al., 2011; Lu et al., 2018). Reduced FA was also detected in the medial and posterior portions of the corpus callosum of children with post-traumatic stress disorder (PTSD; Jackowski et al., 2008), and altered inter-hemispheric frontal, frontal-limbic, or frontal-temporal connectivity was identified as a potential marker of vulnerability to anxiety in young healthy subjects (Yang et al., 2019) and symptomatic anxiety in patients with late-life depression (Li et al., 2020). Finally, it is conceivable that anxiety may lead to elevated blood pressure, a condition that has been associated with scattered ischemic or micro-hemorrhagic white matter lesions occurring in bloodshed regions in older subjects (Iadecola et al., 2016). While this association is unlikely to account for the changes in FA as discussed above, it is worth mentioning that hypertensive patients with dislipidemia showed decreased spectroscopic signal for N-acetylaspartic-acid (NAA), thereby suggesting that associated comorbidities may interfere with the process of new myelin synthesis in white matter tracts (Chiappelli et al., 2019).

## The Importance of Threat and Safety Discrimination in Anxiogenic Mechanisms of Stress- and Anxiety-Related Disorders

In addition to disruptions in threat learning, growing evidence suggests that anxiety disorders are characterized by impaired threat/safety discrimination, resulting in generalized fear that is typically associated with a proliferation of avoidance behaviors that incapacitate daily function and have a negative impact on mental health (Lissek et al., 2009; Sep et al., 2019). Disrupted discrimination derives from several learning processes gone awry: overly strong conditioning to threat, as well as underdeveloped defensive response suppression to non-threatening stimuli. Combined, overactive communication patterns characteristic of making associations between cues and threatening outcomes, and underactive patterns of communication that are characteristic of fear suppression towards non-threatening cues, leads to generalized fear and anxiety (Jovanovic et al., 2010, 2012). Therefore, proper discrimination learning depends on the development and maintenance of connected functional circuits that can support both fear acquisition and fear suppression.

Safety learning is integrally linked to fear learning. Non-threatening neutral cues or safety cues that signal the explicit absence of threat, when learned, become conditioned inhibitors of fear (Pavlov, 1927; Rescorla, 1988). An effective safety cue can also be positively reinforcing because it signals the active lack of threat, and therefore carries motivational and rewarding properties. For example, when presented with a safety cue, animals show increased instrumental responding, such as more vigorous bar pressing for a reward (Hendry, 1967), and stronger conditioned place preference for the area where the safety cue was presented (Rogan et al., 2005). Therefore, learning about non-threatening or safe stimuli is likely to engage a set of regions that are overlapping but distinct from those involved in fear conditioning, and can include circuits that engage the processing of reward (Luo et al., 2018). Although the hippocampal-amygdala-prefrontal network is active during threatening and non-threatening cues, the cell populations involved and modes of communication between these regions are different during these two types of learning (Sangha et al., 2013, 2014; Mayer et al., 2018; Ng et al., 2018). Therefore, encoding modes within and communication between areas are both crucial to maintain accurate and updated information to appropriately dial anxiety, and myelin dysregulation in this circuit has been associated with fear generalization and PTSD (Jovanovic et al., 2010; Fani et al., 2012).

## Circuit-Level Communication That Sculpts Threat-Safety Discrimination Learning

At the circuit level, inter-regional communication underlies the ability to discriminate between threat and safety and is manifested by oscillations that reflect fluctuating membrane potentials in groups of neurons due to incoming inputs from distal sites and local firing (Buzsáki et al., 2013; Akam and Kullmann, 2014; Pesaran et al., 2018). Theta (4–12 Hz) and gamma (30–120 Hz) rhythms are the two main types of oscillations related to discrimination learning. The oscillatory communication patterns between distal sites shift as different regions are exposed to cues that are paired or unpaired with aversive experience (Lesting et al., 2011; Likhtik et al., 2014; Stujenske et al., 2014; Ciocchi et al., 2015; Karalis et al., 2016; Padilla-Coreano et al., 2019).

**Figure 3:**
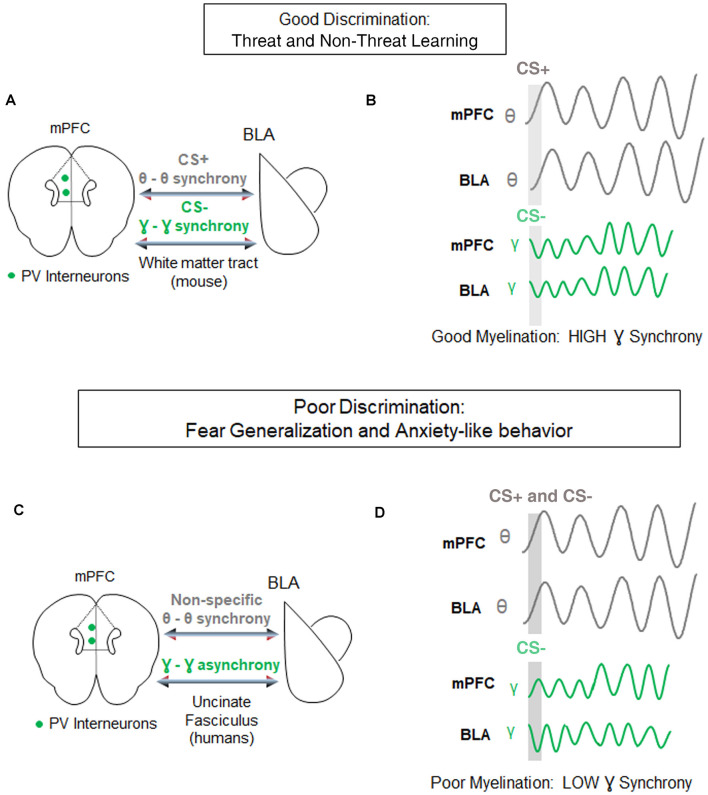
Circuit-level synchrony is required for discrimination of threat from non-threat.** (A)** Under conditions of well-myelinated tracts, medial prefrontal cortex (mPFC)-basolateral amygdala (BLA) communication *via* white matter tracts during successful discrimination of threat from non-threat is characterized by high theta-range synchrony selectively during the aversive CS+ (gray), and high gamma-range synchrony selectively during the discriminated non-aversive CS− (green). Parvalbumin positive (PV) interneurons in the mPFC are posited to contribute to gamma oscillations-based, fast communication during discrimination. **(B)** *Top*, Examples of synchrony in CS+ evoked (gray bar) theta signals of the mPFC and BLA (gray lines). *Bottom*, examples of synchrony in CS− evoked gamma signals of the mPFC and BLA (green lines). **(C)** During poor fear discrimination and compromised myelination, theta synchrony in the mPFC-BLA circuit increases non-selectively to threatening and non-threatening cues (gray), and gamma-synchrony during the non-threatening cues is diminished. The direct communication between these circuits occurs *via* the Uncinate Fasciculus tract in humans. **(D)** The same as in **(C)** but demonstrating non-specific theta-synchrony, and lower gamma synchrony in this circuit during poor discrimination of the CS−.

For instance, during retrieval of differential fear conditioning, presentations of aversive stimuli lead to increased strength and theta synchrony in the basolateral amygdala (BLA) and in the PFC, relative to presentations of the non-threatening stimulus (Likhtik et al., 2014; Karalis et al., 2016). Furthermore, when fear is suppressed during retrieval of non-aversive stimuli, theta oscillations in the PFC predict BLA theta rhythms, suggesting that information from PFC to BLA is transferred *via* oscillatory theta band activity (Likhtik et al., 2014; Figure 3). Likewise, when a fear-conditioned cue becomes less aversive after extinction learning, communication in the theta band decreases between these regions, and PFC theta oscillations organize BLA activity (Lesting et al., 2011; Davis et al., 2017; Rahman et al., 2018), suggesting that theta-encoded information transfer from the PFC to the BLA (carried by the uncinate fasciculus) may be a common signature of fear inhibition across several learning paradigms. Notably, in animals that generalize fear after discrimination learning, theta oscillations remain high in the PFC-BLA pathway during aversive and non-aversive stimuli, without a predominance of theta-information transfer from the PFC to the BLA (Likhtik et al., 2014). Thus, similar PFC-BLA processing of threat and non-threat are signature characteristics of fear generalization.

Gamma oscillations develop along with the maturation of inhibitory signaling and depend on the myelination of Parvalbumin (PV+) GABAergic inhibitory interneurons (Traub et al., 1996; Fries, 2009; Sohal et al., 2009; Hu et al., 2014; Strüber et al., 2015), which are extensively myelinated in an activity-dependent manner (Stedehouder and Kushner, 2017; Stedehouder et al., 2018, 2019). GABAergic activity and gamma oscillations in the PFC are crucial for cue detection and encoding (Courtin et al., 2014; Piantadosi and Floresco, 2014; Howe et al., 2017). While the role of gamma rhythm in fear learning is still the subject of active investigation (Headley and Paré, 2013), fear suppression to non-threat is associated with an increase in gamma-range synchrony in communication among cortical and subcortical regions (Figure 3; Stujenske et al., 2014; Concina et al., 2018). We, therefore, propose that good discrimination between threat and non-threat requires optimal myelination of PV+ interneurons, manifesting in regional gamma synchrony. We further posit that aberrant myelination coupled with impaired white matter integrity of the PFC-BLA connection may result in decreased gamma rhythm, loss of discrimination, and lead to fear generalization.

Furthermore, temporal precision is necessary for inter-regional communication, when BLA gamma oscillations are coupled to PFC theta oscillations during a successfully discriminated non-threat (Stujenske et al., 2014), suggesting that prefrontal input has a direct or indirect role in driving gamma activity in the BLA (Rosenkranz and Grace, 2001; Amano et al., 2010; Bukalo et al., 2015; Strobel et al., 2015; Bloodgood et al., 2018). This faster oscillatory mode of cross-regional communication is shaped by excitatory-inhibitory interactions that require millisecond range timing of inhibitory activity (Buzsáki and Wang, 2012; Courtin et al., 2014). Given its role in speeding up communication, new myelin formation, sheath integrity, and effective remodeling are likely to play an integral role in sculpting inhibitory-excitatory dialogue during discrimination learning.

## Myelin Plasticity and Oligodendrocyte Lineage Cells as Regulatory Mechanisms of Circuit Connectivity

**Figure 4:**
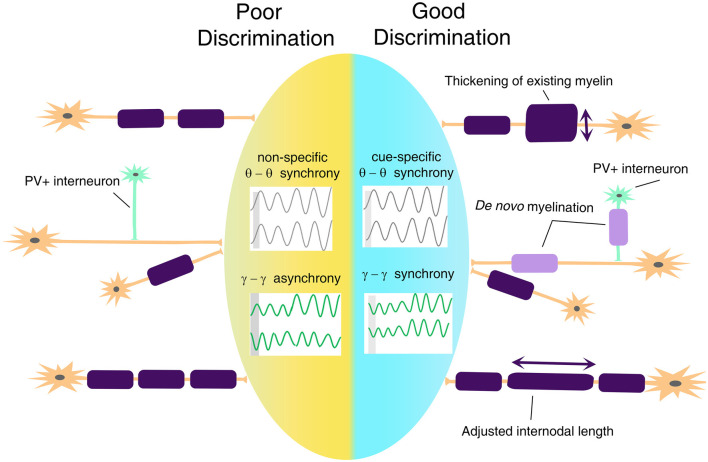
A proposed model for myelin plasticity in discrimination between threat and safety. Myelin remodeling mechanisms include thickening of existing myelin, *de novo* myelination on unmyelinated axons, and adjusted internodal length, which could occur both on excitatory neurons and parvalbumin-positive (PV+) interneurons. Such experience-dependent myelin remodeling allows inter-regional cue-specific theta-range synchrony and gamma-range synchrony to establish, which is essential for successful threat and safety discrimination.

Based on the previously discussed evidence of changes in white matter tracts in anxiety disorders, we posit that disruptions in threat and safety discrimination are related to defective mechanisms of cortical synchronization. Connectivity determines the speed and timing of electrical activity transmitted between relay points, leading to synchronous activation of neural networks and rhythmic oscillations. A mathematical model has predicted that a 1-ms conduction delay would interrupt the phase by 30°, significantly affecting signal amplitude and phase coherence (Pajevic et al., 2014). Besides, myelination may be the most effective way not only of modulating conduction velocity, but also impacting self-organization of brain oscillation and affecting cognitive performance (Mabbott et al., 2006; Scantlebury et al., 2014; Bells et al., 2019; Noori et al., 2020). In terms of threat and safety learning, it will be important to take into consideration the fact that myelination of glutamatergic neurons and PV+ GABAergic interneurons (Stedehouder and Kushner, 2017; Stedehouder et al., 2018, 2019) might bear important and unique functional consequences on the overall activity of the neural networks and consequent oscillations (Figure 4), with altered myelination of PV+ interneurons, likely impacting gamma oscillations and myelination of BLA-mPFC pyramidal tracts impacting theta rhythms.

It is also important to notice that OL lineage cells are capable of influencing neural activity and regulating circuit function in manners that are independent of canonical models of myelin plasticity and myelin remodeling. One example is the role of transmembrane proteoglycan nerve-glia antigen 2 (NG2), which is expressed on the surface of OPCs and has been proposed to act as a neuro-glial signal (Sakry et al., 2014) by being cleaved in an activity-dependent manner and modulating glutamate receptor activity in neighboring neurons (Sakry et al., 2014). Myelinating OLs also regulate K+ homeostasis, due to the expression of inward rectifying K+ channel, K_ir_4.1, and OL-specific conditional ablation of this channel has been linked to delayed recovery of white matter axons from repetitive stimulation (Larson et al., 2018).

Taken together, we, therefore, posit that discrimination between threatening and safe cues may rely on distinct modalities of white matter plasticity or regulation of OL lineage cell function to favor neuronal synchronization across neural networks. We further predict that specific alterations of these mechanisms may be related to the development of anxiety disorders.

## Concluding Remarks

As reviewed above, studying myelination mechanisms in circuits underlying anxious behavior and discrimination learning represents an intriguing approach to understanding the development of anxiety disorders and clarifying novel treatment approaches. To test this framework, it will be important to determine the effectiveness of interventions targeting learning and discrimination processes and their underlying circuit functioning in the treatment of stress- and anxiety-related disorders. The key to this approach will be to understand the multiple ways in which myelin and OL functioning and plasticity contribute to these effects to inform the development of more targeted behavioral interventions that reverse disruptions in circuit-level functioning and ultimately improve management of anxiety symptoms.

## Data Availability Statement

The original contributions presented in the study are included in the article, further inquiries can be directed to the corresponding author/s.

## Author Contributions

All authors conceived the concept and wrote the manuscript. All authors contributed to the article and approved the submitted version.

## Conflict of Interest

The authors declare that the research was conducted in the absence of any commercial or financial relationships that could be construed as a potential conflict of interest.
